# Three Distinct Urethral Fistulae 35 Years After Pelvic Radiation

**DOI:** 10.5812/numonthly.14197

**Published:** 2014-02-22

**Authors:** Arindam Sharma, Michael P. Kurtz, Jairam R. Eswara

**Affiliations:** 1 Government Medical College and Hospital, Chandigarh, India; 2 Massachusetts General Hospital, Harvard Medical School, Boston, USA

**Keywords:** Radiation Injuries, Rhabdomyosarcoma, Surgical Flaps, Urinary Diversion, Urinary Fistula

## Abstract

**Introduction:**

While the development of fistulae is a well-known complication of radiotherapy, such fistulae can often be challenging to manage.

**Case Presentation:**

We describe the case of a 37 year old male who developed in succession a urethrocutaneous fistula to the thigh, a rectourethral fistula and a peritoneo-urethral fistula 35 years after radiotherapy for pediatric pelvic rhabdomyosarcoma. These complications were managed successfully after multiple surgical procedures.

**Discussion:**

We subsequently discuss the different approaches currently employed for the management of radiation induced urinary fistulas and describe the rationale behind our approach towards their surgical management.

## 1. Introduction

Rhabdomyosarcoma is the commonest soft tissue sarcoma among infants and children, accounting for nearly 50% of all cases. Rhabdomyosarcoma of the genitourinary tract accounts for approximately 20% of all cases, with an incidence of 0.5 to 0.7 per million children younger than 15 years (1). The current standard of treatment for rhabdomyosarcoma is a combination of chemotherapy with or without adjuvant radiotherapy and surgical excision. High-dose irradiation (50 to 55Gy) is necessary to ensure local tumor control in patients who are unable to undergo complete surgical resection, even if concomitant multi-agent chemotherapy is given.

 Among the complications of pelvic radiation, development of urinary tract fistulae is rare, with reported incidence of rectourethral fistula varying from 0.2% (2) to 1% (3), There have been only 2 case reports of urethrocutaneous fistula following radiation (4, 5). Although some urinary fistulae heal with conservative management, surgery is often necessary for definitive management.

## 2. Case Presentation

**Figure 1.**
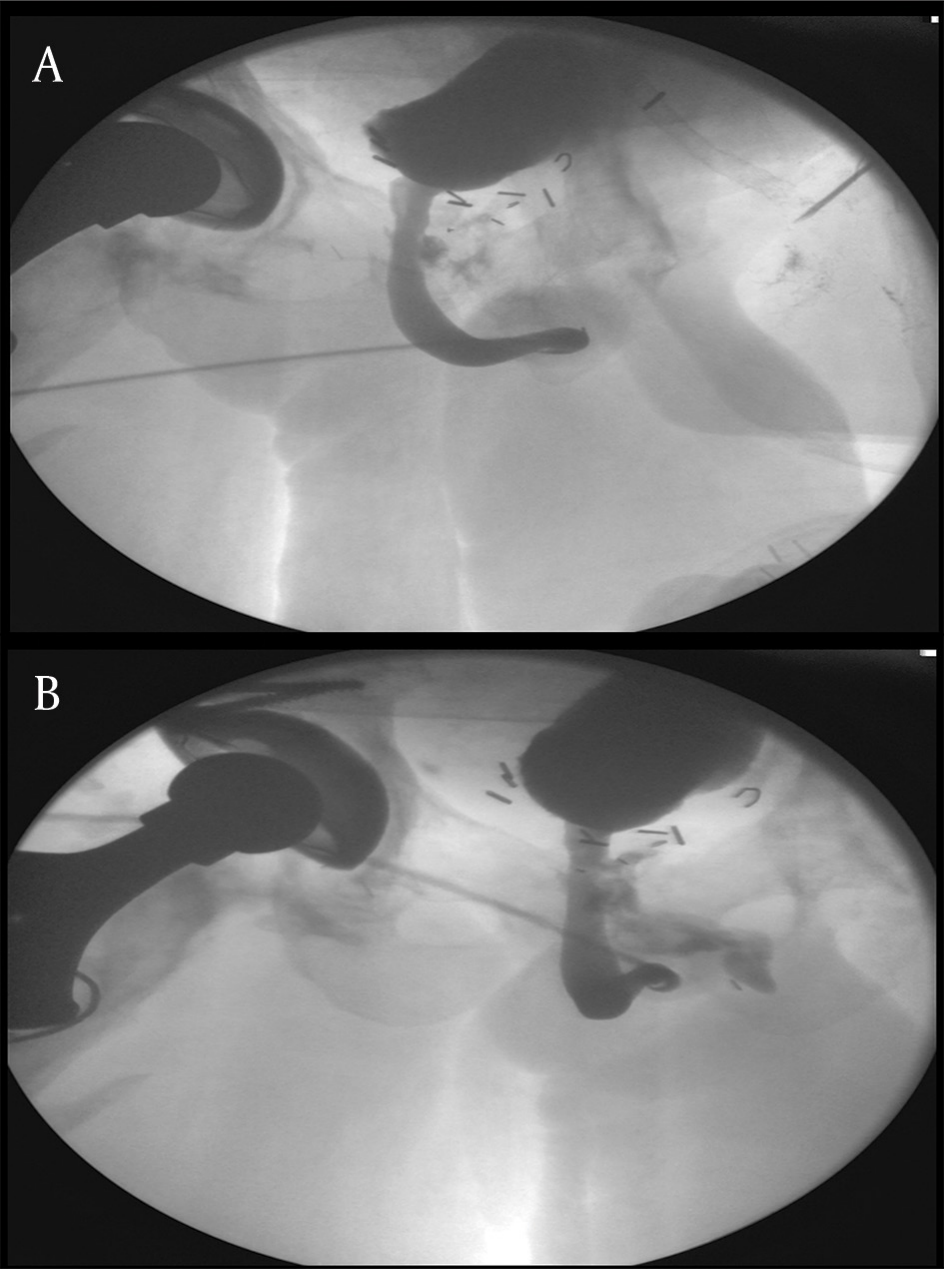
Retrograde Urethrogram Performed After Patient Developed Leakage of Urine From Left Thigh a) Leakage of contrast from bulbar urethra, b) Urethrocutaneous fistula from urethra to left thigh demarcated with contrast

A 37 year old male and current smoker (1 pack per day) with a history of prostate rhabdomyosarcoma at age 2 treated with partial resection and high dose external beam radiation presented to the emergency department (ED) with fever, groin pain and abdominal distension. His history was notable for numerous complications including bilateral osteonecrosis of the hip, lifelong complete urinary incontinence, a dense rectal stricture causing chronic colonic dilation and critical lower extremity ischemia due to bilateral iliofemoral disease. He had undergone 45 procedures to address these complications. He was initially managed conservatively for intestinal obstruction secondary to a rectal stricture. Rectal dilation was performed. Subsequently, he developed MSSA (methicillin sensitive *Staphylococcus aureus*) bacteremia and an abscess was noted in his left thigh (adductor region). The abscess was drained under ultrasound guidance by the interventional radiology team, and the drain was removed after a week. A month later, he presented to the ED with fluid draining from the former site of the IR drain. The fluid was found to be urine, with creatinine of 30.9 mg/dL. A Foley catheter was placed, and retrograde urethrogram (RUG) showed a urethrocutaneous fistula from the bulbar urethra to the left thigh (Figure 1). Cystoscopy and subsequent biopsy of the fistula revealed no malignancy. Given the need of serial rectal dilations for his chronically dilated large bowel, the patient was formally recommended a permanent colostomy, but he declined. He was discharged with catheter in place and the fistula site dry.

**Figure 2.**
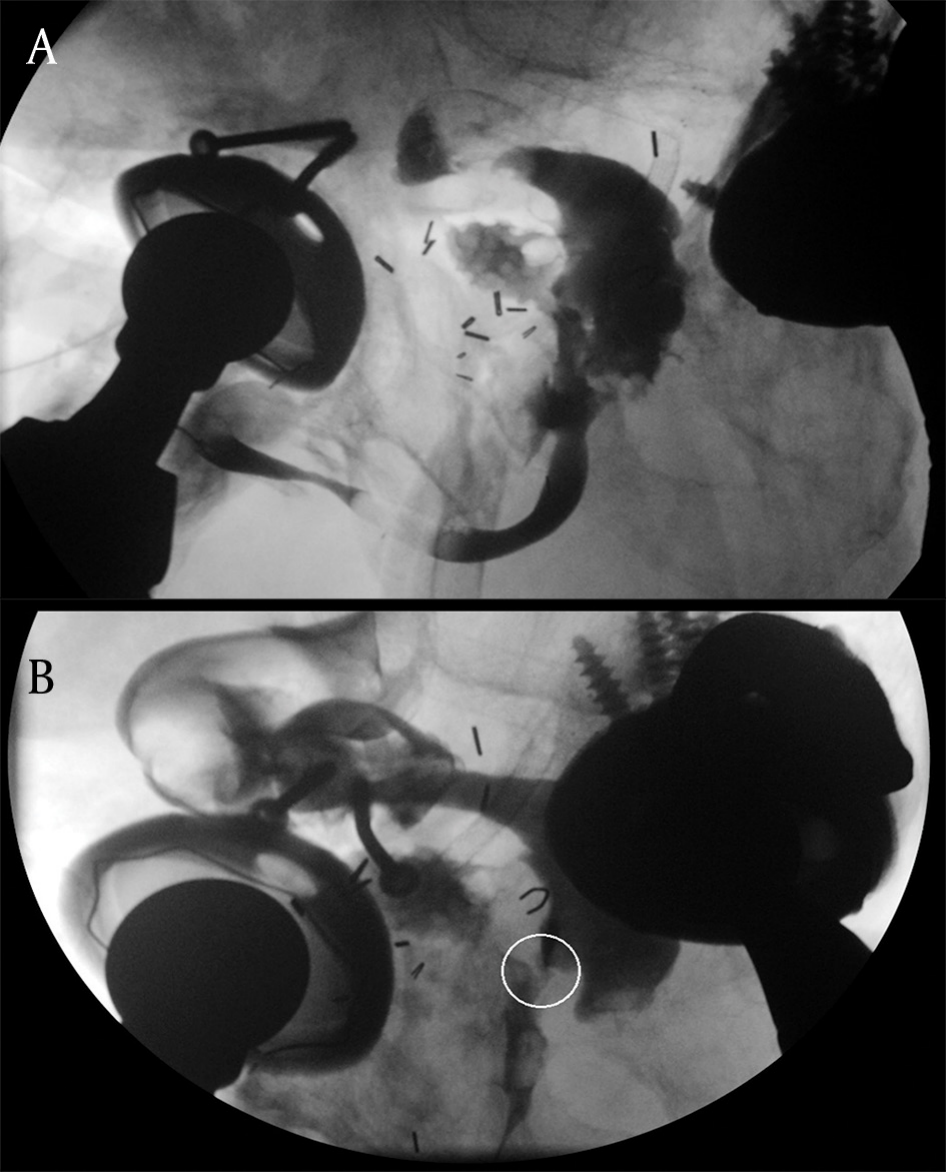
Retrograde Urethrogram Performed After Patient Developed Leakage of Stool From Left Thigh a) Leakage of contrast from urinary tract into the rectum, b) Rectourethral fistula (encircled) demarcated with contrast

As definitive treatment for the fistula, an ileal conduit urinary diversion with partial cystectomy was done. The patient was discharged postoperatively on day 12, with fistula dry and scant discharge per urethra. Two months after this surgery, the patient developed a cold lower limb for which he underwent a successful axillary-femoral artery graft thrombectomy. The next month, he also underwent a major hip joint reconstruction. 8 weeks later, he developed fever and chills followed by passage of feces per urethra. The next week, he developed fecal drainage from the left thigh as well. A RUG was performed and revealed a rectourethral fistula (Figure 2). To treat this rectourethral fistula an end colostomy with distal mucus fistula was performed. Drainage from the urethra and thigh fistula persisted despite the surgery. Proctoscopy showed a tight rectal stricture at 8 cm. Colonic irrigation through a red rubber tube, passed through the distal mucus limb of his colostomy was done with the aim of clearing out the stool in his distal colon from his thigh fistula.

The cutaneous fistula remained dry for several weeks. Leakage per rectum remained minimal. He then reported passage of sutures per urethra and discharge of straw-colored peritoneal fluid from both the urethra and thigh fistulae. As a temporary measure, he underwent intraoperative cystoscopic identification of the fistula site and urethral bulking therapy using a dextranomer and hyaluronic acid (Deflux®, Salix Pharmaceuticals, Raleigh) applied to the fistula site. Debridement of the left groin fistula tract, and negative pressure wound therapy placement to control drainage from the peritoneo-urethral fistula was also done. Postoperatively, the drainage from the thigh and urethra persisted, so he underwent takedown of the previous descending colostomy and mucus fistula, descending colectomy and proctectomy, creation of a new permanent colostomy and simple cystectomy. As drainage still persisted after this, his urethra was closed perineally proximal to the fistula, and the defect was reinforced with a pedicled rectus femoris muscular flap. His incisions have healed well, and he no longer has drainage 5 months after surgery.

## 3. Discussion

This case is an example of late-onset radiation injury, with rectal and genitourinary complications 35 years after radiation. Radiation-induced fibrosis is the hallmark of such injury. Acute inflammation at the time of radiation initiates a fibrogenic cascade similar to that seen in normal tissue injury and scarring. The resultant fibrogenic response is mediated by several cytokines especially TGF-beta, PDGF, and IL-13. This fibrogenic cascade is dysregulated and altered in irradiated tissues, resulting in pathological fibrosis and its complications including strictures and fistulae (6, 7). Risk factors such as cigarette smoking, race, and increased body mass index have been associated with major late complications of the bladder, rectum and small bowel following pelvic radiation (8, 9). Though long considered irreversible, promising therapies using agents such as pentoxifylline, vitamin E, clodronate, superoxide dismutase and hyperbaric oxygen have begun to show that radiation-induced fibrosis may be preventable and reversible (10-12).

The debate on whether a radiation induced fistula deserves an attempt at repair or be dealt aggressively with permanent diversion surgeries from the start is presently unsettled. In their study, Linder et al. (13) advocate that permanent urinary diversion with our without permanent colostomy should be considered early in the surgical management of these patients. They reported that primary repair was successful in only 1 out of 6 patients with radiation induced fistulas in whom primary repair was attempted, compared to successful repair in 15 out of 16 patients with fistulas due to other causes. A vast majority of patients with radiation induced fistulas required permanent colostomy (25/29), or permanent urinary diversion (27/29). On the other hand, Lane et al. (14) report in their study that out 18 patients with radiation induced rectourethral fistulas taken up for definitive surgery, successful fistula repair was performed without any form of permanent diversion in 8 patients ,and repair with preservation of at least one sphincter was performed in 5 patients. Combined fecal and urinary diversion was required only in 5 patients. Successful fistula repairs in the setting of radiation have also been reported by Voelzke et al. (15) ( n = 13, 8 successful), Vanni et al. (16) (n = 39, 84% repaired in single stage), Lesser et al. (17) and Iachetta et al. (18).

The term 'Radiation Induced Fistula' is a blanket term, encompassing a heterogeneous group of patients having diverse variations in fistula anatomy, pre-morbid urinary and bowel function, health of surrounding tissues, presence of co-morbidities, life expectancy, and severity of radiation exposure Each of these factors can affect surgical outcomes. Thus, creating and defining treatment protocols based upon superficial categories like 'radiation induced' vs. 'non radiation induced' types of fistulas is unlikely to provide optimum decisions for patients.

Therefore, we stress upon the need to approach such patients on a highly individualized, case by case basis, giving due importance to individual factors and patient preferences. Initial management of radiation induced urinary fistulae involves urinary and/or fecal diversion. Further definitive management of the fistula should be decided on the basis of patient factors such as life expectancy, pre-morbid urinary and bowel function, continence, local anatomy and the technical feasibility of a repair (14). Thus, cystoprostatectomy and ileal conduit urinary diversion is appropriate in a patient lacking bladder control, whereas a continent patient would benefit from a urethral repair along with muscle/omentum interposition. Likewise, pre-operative fecal continence would influence the choice between a primary bowel repair or a proctocolectomy and permanent colostomy. Of prime importance is the patient’s personal preference, and the risks and benefits of diversion versus repair should be extensively discussed with the patient to assist them in making an appropriate decision.

Our patient, apart from his urethral fistulas, was also suffering from several other co-morbidities which were of great concern to him, including an extremely painful hip joint that needed reconstruction. He had already undergone 45 major surgical procedures to repair his pelvic and inguinal vasculature and his hip joints. The absence of urinary continence since childhood, and the need for total clearance of infection from the surrounding tissues in order to allow the patient to proceed for an orthopedic hip reconstruction also favored in the decision to perform permanent ileal conduit urinary diversion. The patient declined to undergo permanent colostomy as treatment for his rectal strictures. To treat the rectourethral fistula that subsequently developed temporary fecal diversion was performed. An end colostomy with distal mucus fistula was preferred over a simple loop colostomy since his descending colon was dilated to such an extent that a loop colostomy would have required a very large abdominal stoma, with its inherent complications. As this too failed to control discharge, permanent colostomy, with complete cystectomy was finally agreed upon and performed.

Given the inability of the tissues to hold sutures which resulted in the peritoneo-urethral fistula, urethral bulking using Deflux™ (a Dextranomer and Hyaluronic Acid polymer) was done. It was extensively discussed with the patient that while urethral bulking could diminish the flow, but it was not expected to provide definitive cure, and could be thought of as the first step of the reconstructive process. One of the indications for urethral bulking therapy is incontinence associated with late radiation effects on the bladder and urethra. These cases involve radiation-induced detrusor are flexia, poor bladder compliance, moderate residual urine, constant urinary leakage and a rigid, open, nonfunctional urethra. Open surgery will not cure such incontinence, and might elevate the detrusor leak-point pressure and cause upper-tract disease. Bulking agents do not change detrusor leak-point pressures and are, therefore, safer in these circumstances (19, 20). Complete control of the peritoneo-urethral fistula was obtained by closing down his urethra perineally proximal to the fistula, and the reinforcement of the defect with a pedicled rectus femoris muscular flap.

Radiotherapy related complications should always be kept in mind while treating patients with a past history of radiation, as shown in this case where urological complications started 35 years after pelvic radiation. Individual factors must be taken into account while making the decision between permanent urinary/fecal diversion and definitive repair. Since attempts at repair in such cases carry a substantial risk of failure and need for repeat surgeries, there is need for studies to further stratify such patients according to their individual characteristics. Surgical management of urinary fistulae in the setting of radiation should be approached on a case by case basis, because repair may involve innovative or improvisational maneuvers in the operating room.
